# Characterize the Growth and Metabolism of *Acidithiobacillus ferrooxidans* under Electroautotrophic and Chemoautotrophic Conditions

**DOI:** 10.3390/microorganisms12030590

**Published:** 2024-03-15

**Authors:** Quansheng Wang, Haijun Long, Huiqi Wang, Maggie C. Y. Lau Vetter

**Affiliations:** 1 Laboratory of Extraterrestrial Ocean Systems (LEOS), Institute of Deep-sea Science and Engineering, Chinese Academy of Sciences, Sanya 572000, China; wangqs@idsse.ac.cn (Q.W.); longhj@idsse.ac.cn (H.L.); wanghq@idsse.ac.cn (H.W.); 2 University of Chinese Academy of Sciences, Beijing 101408, China

**Keywords:** acidophile, *Acidithiobacillus ferrooxidans*, electroautotrophic, chemoautotrophic, transcriptome, metabolome

## Abstract

Acidophiles are capable of surviving in extreme environments with low pH. *Acidithiobacillus ferrooxidans* is a typical acidophilic bacterium that has been extensively studied when grown chemoautotrophically, i.e., when it derives energy from oxidation of Fe^2+^ or reduced inorganic sulfur compounds (RISCs). Although it is also known to grow with electrons supplied by solid electrodes serving as the sole source of energy, the understanding of its electroautotrophic growth is still limited. This study aimed to compare the growth characteristics of *A. ferrooxidans* under electroautotrophic (ea) and chemoautotrophic (ca) conditions, with an attempt to elucidate the possible mechanism(s) of extracellular electron flow into the cells. Jarosite was identified by Raman spectroscopy, and it accumulated when *A. ferrooxidans* used Fe^2+^ as the electron donor, but negligible mineral deposition occurred during electroautotrophic growth. Scanning electron microscopy (SEM) showed that *A. ferrooxidans* possesses more pili and extracellular polymeric substances (EPSs) under electroautotrophic conditions. A total of 493 differentially expressed genes (DEGs) were identified, with 297 genes being down-regulated and 196 genes being up-regulated in ea versus ca conditions. The genes known to be essential for chemoautotrophic growth showed a decreased expression in the electroautotrophic condition; meanwhile, there was an increased expression of genes related to direct electron transfer across the cell’s outer/inner membranes and transmembrane proteins such as pilin and porin. Joint analysis of DEGs and differentially expressed metabolites (DEMs) showed that galactose metabolism is enhanced during electroautotrophic growth, inducing *A. ferrooxidans* to produce more EPSs, which aids the cells in adhering to the solid electrode during their growth. These results suggested that electroautotrophy and chemoautotrophy of *A. ferrooxidans* have different extracellular electron uptake (EEU) pathways, and a model of EEU during electroautotrophic growth is proposed. The use of extracellular electrons as the sole energy source triggers *A. ferrooxidans* to adopt metabolic and subsequently phenotypic modifications.

## 1. Introduction

Acidophilic bacteria are important extremophilic microorganisms and are widely used in industrial bioleaching of copper and other sulfide ores due to their ability to thrive in extreme conditions [1,2,3]. Bioleaching mediatedby microbiomesis believed to account for over 30% of global copper production from low-grade copper ores [4]. *Acidthiobacillus* spp., in particular, have evolved to derive energy from iron and/or sulfur oxidation in low-pH environments (<5) [5]. This ability plays a crucial role in the biogeochemical cycling of iron and sulfur and has significant biotechnological potential in emerging applications such as electronic waste cycling [6,7]. The physiological and metabolic characteristics of these bacteria have attracted considerable attention due to their unusual nature. Research has focused on understanding the fundamental biology of extreme microbial adaptations and exploiting these organisms as a basis for biotechnological applications.

*A. ferrooxidans* is a well-studied acidophilic bacterium that belongs to the phylum *Pseudomonadota* (previously *Proteobacteria*) and class *Acidithiobacillia*. It was first isolated from acid mine wastewater in 1951 by Colmer and was originally named *Thiobacillus ferrooxidans* [8]. This Gram-negative bacterium grows optimally at pH 2.0 and 30 °C [9]. *A. ferrooxidans* is a facultatively anaerobic chemolithoautotrophic bacterium that uses O_2_ as an electron acceptor to fix atmospheric CO_2_ via the Calvin–Benson–Bassham (CBB) cycle [10]. It derives energy from the aerobic oxidation of Fe^2+^ or reduced inorganic sulfur compounds (RISCs) [11,12]. Additionally, it can survive under anaerobic conditions with Fe^3+^ as an electron acceptor and RISCs as an electron donor through dissimilatory iron reduction [13]. Hydrogen, formic acid, uranium, and electric current are recognized as alternative energy sources for its growth and respiration [14,15]. *A. ferrooxidans* can be found in natural environments rich in iron and RISCs, such as coal seams, metal–sulfide ores, seawater, and acid mine drainage [16], contributing significantly to the biogeochemical cycling of metals and nutrients. *A. ferrooxidans* plays a crucial role in the industrial bioleaching or biomineralization of metal–sulfide minerals. In addition, *A. ferrooxidans* can survive in Mars-like conditions, making it an interesting model organism for astrobiological studies [17,18,19]. It thrives in extremely acidic environments and is commonly used as a model organism for adaptation studies. Studying *A. ferrooxidans* will also aid in the comprehension of extremophilic microorganisms.

In the iron-oxidizing pathway, *A. ferrooxidans* oxidizes Fe^2+^ to Fe^3+^ with outer-membrane cytochrome c (Cyc2). Electrons then flow toward rusticyanin (Rus) before branching off onto either the uphill or downhill electron transport pathways [20]. To ensure the survival of *A. ferrooxidans*, it is necessary to synthesize sufficient ATP through the downhill pathway [21]. It has been reported that approximately 95% of these electrons enter a downhill channel that is composed of a series of electron transport carriers [16]. The downhill pathway for electron transfer is as follows: Fe^2+^ → Cyc2 (on outer membrane) → Rus → Cyc1 (cytoplasm) → Cox (aa3) → O_2_ [22,23,24]. To maintain reducing power for cell metabolism, approximately 5% of the electrons enter the uphill pathway during the process of Fe^2+^ oxidation and are transferred by a series of electron transport carriers that ends with NA(P)D^+^, which is reduced to NAD(P)H by NAD(P)H reductase [25]. The uphill pathway can be described as Fe^2+^ → Cyc2 (on outer membrane) → Rus → CycA (cytoplasm) → bc1 (cytoplasmic membrane) → quinone → reductase Q → NA(P)D^+^ [26].

Research has demonstrated that *A. ferrooxidans* can function as a biocathode in bioelectrochemical systems [27]. Additionally, it has the ability to directly absorb electrons from solid electrodes [28] and utilize electrons as the exclusive energy source for O_2_ reduction and CO_2_ fixation [14,29]. Ishii and coworkers proposed that the uphill or downhill electron transport pathways of *A. ferrooxidans* are also active under electroautotrophic conditions [14]. However, knowledge is lacking about how the metabolism and growth of *A. ferrooxidans* under the less-known electroautotrophic condition differs from those under the better-understood chemoautotrophic conditions. Understanding such differences, especially the mechanism(s) of electron acquisition, would provide insights into the adaptation/survival strategies of *A. ferrooxidans* and other organisms that are able to switch between chemical and electrical energy and thus facilitate the development of their bioprospect applications.

To expand our view on the growth and metabolic characteristics of *A. ferrooxidans* under electroautotrophic and chemoautotrophic conditions, we compared and contrasted the phenotypic, transcriptomic, and metabolic features of *A. ferrooxidans* grown with these two modes of nutrition, with a focus on understanding how the bacterium acquires energy from different electron sources. The results indicated that the electroautotrophic and chemoautotrophic growth of *A. ferrooxidans* are regulated differently; electron acquisition through chemoautotrophic growth of *A. ferrooxidans* is mediated primarily by outer-membrane cytochrome c via uphill/downhill electron transport pathways, but the electroautotrophic growth of *A. ferrooxidans* appears to rely more heavily on pilin and transmembrane proteins on the cell membrane for extracellular electron uptake, which has not previously been reported in the literature.

## 2. Materials and Methods

### 2.1. Bacterial Strain and Growth Medium

The type strain of *A. ferrooxidans* DSM 14882 (or ATCC 23270 or NCIMB 8455) was obtained from the DSMZ-German Collection of Microorganisms and Cell Cultures GmbH, Leibniz Institute, Germany. Cells were activated and maintained by culturing chemoautotrophically in 9K medium, which consisted of solution A (0.1 g/L KCl, 0.5 g/L MgSO_4_·7H_2_O, 3.0 g/L (NH_4_)_2_SO_4_, 0.5 g/L K_2_HPO_4_, 0.01 g/L Ca(NO_3_)_2_ and solution B (44.2 g/L FeSO_4_·7H_2_O) [30]. The pH was adjusted to 1.8 with 5M H_2_SO_4_ [31]. *A. ferrooxidans* was cultured at 30 °C and 150 rpm. For electroautotrophic cultivation, only solution A was used (i.e., Fe^2+^-free) and without shaking.

### 2.2. Setup of Electrochemical Cultivation

To grow *A. ferrooxidans* electroautotrophically, a bioelectrochemical system was set up referencing that described in the study by Ishii and coworkers [14]. Briefly, a single-chamber three-electrode system (volume 5 mL, Gaoss Union, Wuhan, China) was used, with the working electrode placed on the bottom of the reactor. The working electrode was a fluorine-doped tin oxide (FTO)-coated glass electrode, with a dimension of 30 × 30 × 2.2 mm and a resistance of 15 Ω. The effective surface was a circle with a geometric area of 0.8 cm^2^. The reference and counter-electrodes were Ag/AgCl (KCl sat.) and a platinum wire, respectively. The electrochemical cell was equipped with a transparent base plate for fixing the electrodes and facilitating in situ optical observation.

The pH of solution A was adjusted to 1.8 using 5 M H_2_SO_4_. The headspace of the reactor was purged with air, which provided the source of O_2_ and CO_2_. The voltage was set to +0.4 V vs. standard hydrogen electrode (SHE), and an amperometric i-t curve was used to monitor the change of current over time. Via linear sweep voltammetry [14], the onset potential of the cathodic current for the cells attached to the electrode as the electric conduit in solution A (+0.33 V vs. SHE) was verified to be different from that of the Fe^3+^/Fe^2+^ in the electric conduit in 9K medium (+0.20 V vs. SHE). The electrochemistry workstation used in this study was CHI1040C model (CH Instruments, Inc., Bee Cave, TX, USA). All experiments were performed at 30 °C. Prior to first use, the electrochemical apparatuses were conditioned. The conductive FTO glass electrode was cleaned in an ultrasonic bath using toluene for 10–20 min, followed by acetone for 10–15 min, then ethanol for 10–20 min, and lastly deionized water for 20–30 min; the glass electrode was preserved in absolute ethanol for long-term storage [32]. The platinum counter-electrode, the KCl-saturated Ag/AgCl reference electrode, and the electrolytic cell as a whole were rinsed with 75% ethanol, and then deionized water three times. After each experiment, the electrodes and the whole electrolytic cell were cleaned by submerging them in 75% ethanol for 30 min, and then air-dried while exposed to UV light for 30 min and stored in enclosed containers.

### 2.3. Cell Growth Monitoring and Cell Concentration Measurements

It is common and convenient to monitor cell growth by optical density, e.g., OD600 for *Escherichia coli*. To identify whether there is a diagnostic wavelength for monitoring changes in the number of chemoautotrophic *A. ferrooxidans* cells, a full scan across 200–900 nm was performed, and a maximum absorption peak at 420 nm was consistently observed for cells collected from the exponential and plateau phases. Therefore, the cell density of chemoautotrophic cells was estimated by measuring the change in absorbance at 420 nm.

For chemoautotrophic growth experiments, bacteria in the logarithmic growth phase were inoculated into three 200 mL conical flasks with 150 mL liquid medium at a 10% *v*/*v* ratio. Triplicate aliquots were taken out every 12 h for determining the optical density as described above. At each sampling point, 200 μL of the chemoautotrophic bacterial culture solution was extracted and added to a 96-well cell optical plate for OD420 measurement using a plate-reader (SpectraMax ID3, Sunnyvale, CA, USA). Absorbance was measured immediately after inoculation (i.e., T = 0) until no significant change was observed (i.e., having reached the plateau phase). A blank control group was established using 9K liquid medium (as the control group). Optical observation of *A. ferrooxidans* was conducted using a 3D holographic microscope (Nanolive 3D Cell Explorer, Tolochenaz, Switzerland), and cell counting was performed using a bacterial cell counting plate (HD-825, THOMA, Beijing, China). The depletion of Fe^2+^ was detected using potassium dichromate titration, following the method outlined in 2015 [33]. The data was used to construct the growth curve, which was expressed as the mean and standard error of three replicates.

For electroautotrophic growth experiments, chemoautotrophic cells were harvested from the logarithmic growth phase (48~72 h) by centrifugation at 7200 rpm for 10 min, and washed at least three times with solution A (pH 1.8). The washing step was performed to eliminate soluble Fe^2+^ ions and insoluble iron oxides from the cell culture. Washed cells were diluted to OD420 = 0.02 and inoculated into the BES described in Section 2.2. Cell density was determined by cell counting as described above for chemoautotrophic cells.

### 2.4. Scanning Electron Microscopy and Raman Spectroscopy

Electroautotrophic and chemoautotrophic cells in the logarithmic growth phase were collected by centrifugation at 7200 rpm for 10 min, rinsed three times with 1 mL of pH 7.0 PBS, and fixed in a final concentration of 5% glutaraldehyde solution. Precipitates were observed during chemoautotrophic growth and were sampled from the bottom of the conical flask, then were dehydrated at room temperature. The fixed cells and precipitates were analyzed using SEM (without gold coating). Micro-structures and the main elements were evaluated using a Philips XL-30 SEM equipped with a secondary electron detector and an energy-dispersive X-ray spectrometer (EDS). The SEM was operated at an accelerating voltage of 2.0 kV with a working distance of 5–10 mm, and images were captured in secondary electron imaging mode. An accelerating voltage of 10 kV was used for EDS analysis to obtain sufficient X-ray counts for the generation of local elemental maps [34].

Raman spectroscopy was performed using the Alpha 300R spectrometer (WITec GmbH, Ulm, Germany) to identify precipitates collected from the chemoautotrophic growth experiment. The precipitates were taken from the culture medium and placed on a glass slide, dehydrated at room temperature, and covered with a cover slide. Raman spectra were obtained by focusing the laser beam on the specimen beneath the surface to avoid potential contamination and artifacts on surfaces [35,36]. The samples were irradiated with a 532 nm laser with a power of 0.2 mW. Scattered light of 150–3800 cm^−1^ was collected by a microscope (Zeiss, Oberkochen, Germany) at 100× magnification, with integration time of 5 s. Data processing was performed using Project Five version 5.3 Plus. Comparison and identification of Raman spectra were referred to RRUFF database (https://rruff.info/; accessed on 25 September 2022).

### 2.5. RNA Extraction, Library Preparation, and Sequencing

Three replicates of electroautotrophically grown cells served as the experimental group whereas three replicates of chemoautotrophically grown cells served as the control group, hereafter referred to as ‘ea’ and ‘ca’, respectively. RNA was extracted from a total of 50 mg of *A. ferrooxidans* cells (ea, T = 48 h; ca, T = 60 h) using TRIzol^®^ Reagent (Thermo Fisher, Waltham, MA, USA) according to the manufacturer’s instructions. RNA quality was assessed using a 5300 Bioanalyser (Agilent, Santa Clara, CA, USA) and quantified with nanodrop (ND-2000, NanoDrop Technologies, Wilmington, DE, USA). Only high-quality RNA samples (OD_260/280_ = 1.8~2.2, OD_260/230_ ≥ 2.0, RIN ≥ 6.5, 28 S:18 S ≥ 1.0, quantity >1 μg) were used for library construction. Small-RNA libraries were generated following the manufacturer’s recommendations using the QIAseqmiRNA Library Kit (Qiagen, Hilden, Germany). The RNA libraries were generated using the Illumina Stranded Total RNA Prep with Ribo-Zero Plus and were sequenced using an Illumina NovaSeq 6000 (Illumina, San Diego, CA, USA). The method described here was carried out at Majorbio Bio-pharm Biotechnology Co., Ltd. (Shanghai, China). A total of 3 ea and 3 ca blank cultivation controls were processed in the same manner as their cell-containing counterparts, and no detectable RNA was extracted, and therefore, no RNA library was constructed for sequencing.

### 2.6. Transcriptomic Analysis

The sequences were processed and analyzed using the bioinformatic pipeline provided by Majorbio Bio-pharm Biotechnology Co., Ltd. (Shanghai, China). Briefly, to obtain high-quality reads, the paired-end reads obtained from the *A. ferrooxidans* ea and ca groups underwent adapter removal and quality analysis using CASAVA (v1.8.2). Paired-end reads with an N exceeding 10% of the individual read length were considered low-quality sequences and filtered out. All high-quality reads had Q30 scores of 95% or higher, providing high-quality sequencing reads for downstream analysis. The high-quality sequencing reads after splicing (unigenes) were then annotated and classified using various databases, such as the National Center for Biotechnology Information (NCBI) non-redundant (nr), Swiss-Prot, Pfam, KEGG, Gene Ontology (GO), and Clusters of Orthologous Groups of proteins (COGs), with the aid of BLAST (v2.12.0).

High-quality reads of each of the 6 samples were aligned to the transcripts using Botwie2, and gene expression was calculated using RNA-seq by expectation maximization (RSEM). The expression level of each unigene was calculated by normalizing to the TPM (transcripts per million reads) value of the transcript containing the unigene. To identify differentially expressed genes (DEGs), DEseq2 package (v1.22.2) in R was used to analyze the data between two groups, based on a false discovery rate of <0.05 and an absolute log_2_ fold-change [37] of ≥1 [13].

### 2.7. Metabolite Extraction and UHPLC-MS/MS Analysis

Six replicates of ‘ca’ samples and six replicates of ‘ea’ samples were prepared and sent to Majorbio Bio-pharm Biotechnology Co., Ltd. (Shanghai, China) for extraction and data acquisition. The specific steps were performed as described in 2022 [38]. Briefly, 50 mg of *A. ferrooxidans* cells and 6 mm-diameter grinding beads were placed in a 2 mL Eppendorf tube. For extracting metabolites, 400 μL of extraction buffer (methanol: water = 4:1 *v*/*v*) containing 0.02 mg/mL of internal standard (L-2-chlorophenylalanine) was used. The samples were ground using a Wonbio-96c frozen tissue grinder (Shanghai Wanbo Biotechnology Co., Ltd., Shanghai, China) for 6 min at −10 °C and 50 Hz, followed by low-temperature ultrasonic extraction for 30 min at 5 °C and 40 kHz. The samples were stored at −20 °C for 30 min, then centrifuged for 15 min at 13,000× *g* at 4 °C. The resulting supernatant was transferred to the injection vial for ultra-high pressure liquid chromatography-tandem mass spectroscopy (UHPLC-MS/MS) analysis.

UHPLC-MS/MS analysis of the metabolite extracts was performed using a Thermo Scientific Q Exactive HF-X system equipped with an ACQUITY HSS T3 column (100 mm × 2.1 mm i.d., 1.8 μm; Waltham, MA, USA). The mobile phases consisted of 0.1% formic acid in water:acetonitrile (95:5, *v*/*v*) (solvent A) and 0.1% formic acid in acetonitrile:isopropanol:water (47.5:47.5:5, *v*/*v*) (solvent B). The flow rate was 0.40 mL/min, and the column temperature was maintained at 40 °C.

For mass spectrometry, an electrospray ionization (ESI) source was operated in positive and negative modes. The optimal conditions were set as follows: the source temperature was set to 425 °C, the sheath gas flow rate was set to 50 arb, the Aux gas flow rate was set to 13 arb, and the ion-spray voltage floating (ISVF) was set to -2800 V in negative mode and +3500 V in positive mode, respectively. Normalized collision energy was set to 20–40–60 V rolling for MS/MS. The full MS resolution was 60,000, and the MS/MS resolution was 7500. Data acquisition was performed using the data-dependent acquisition (DDA) mode, and detection was carried out over a mass range of 70–1050 mass/charge ratio (*m*/*z*).

### 2.8. Metabolomic Analysis

The UHPLC-MS/MS raw data was pretreated using Progenesis QI (Waters Corporation, Milford, CT, USA) software. The software exported a three-dimensional data matrix, which includes sample information, metabolite names, and mass spectral response intensity, in CSV format. The metabolites were identified by searching against the databases such as the Human Metabolome Database (HMDB, http://www.hmdb.ca/; accessed on 15 September 2023), the Metabolite and Chemical Entity Database (METLIN, https://metlin.scripps.edu/; accessed on 15 September 2023), and the Majorbio Database [39].

The resulting data matrix was uploaded to the Majorbio cloud platform (https://cloud.majorbio.com; accessed on 20 September 2023) for analysis. The data matrix was pre-processed to retain at least 80% of the metabolic features detected in any set of samples. After filtering for samples with metabolite levels below the lower limit of quantification, the minimum metabolite value was estimated, and each metabolic signature was normalized to the sum of individual sample. The final data matrix for subsequent analysis excluded variables of QC samples (internal standard) with a relative standard deviation (RSD) greater than 30%, which were logarithmicized using log_10_.

Principal component analysis (PCA) and orthogonal least partial squares discriminant analysis (OPLS-DA) were performed using the R package ‘ropls’ (v1.6.2), and the stability of the model was evaluated using 7-cycle interactive validation. Based on the variable importance in the projection (VIP) obtained by the OPLS-DA model and the *p*-value generated by Student’s *t*-test, metabolites with VIP > 1 and *p* < 0.05 were considered significantly different between the ca and ea groups.

Differential metabolites between the ca and ea groups were mapped to their respective biochemical pathways using metabolic enrichment and pathway analysis based on the KEGG database (http://www.genome.jp/kegg/; accessed on 25 September 2023). The metabolites could be classified based on their involved pathways or performed functions. The KEGG enrichment analysis to obtain the most relevant biological pathways for experimental treatments was performed using the Python package ‘scipy.stats’ (https://docs.scipy.org/doc/scipy/; accessed on 25 September 2023).

### 2.9. Joint Analysis of Transcriptome and Metabolome

The joint analysis was performed using the online Majorbio cloud platform (https://cloud.majorbio.com/; accessed on 20 November 2023). The parameters used the default settings. The software programs used were as follows: scipy (Python) (Version 1.0.0), OmicsPLS (R packages) (Version 2.0.2), vegan (R packages) (Version 2.6.4), KEGG (Version v20221012), iPath (Version 3.0).

## 3. Results and Discussions

### 3.1. Avoiding Lausenite Formation in 9K Medium

**Figure 1:**
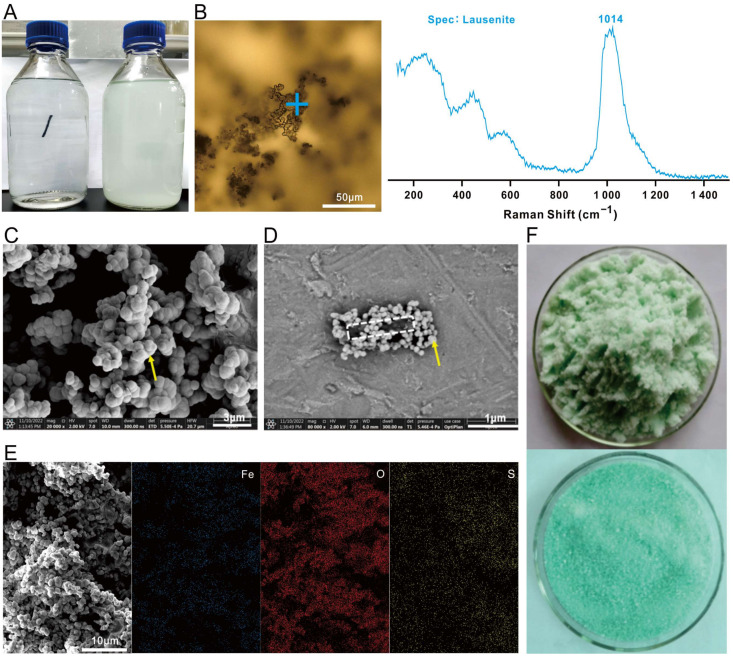
Precipitation of lausenite during the preparation of the 9K medium. (**A**) Clear 9K medium (left) verses cloudy 9K medium (right). (**B**) Microscopic picture of precipitates under brightfield, with the blue ‘+’ indicating the Raman laser spot center and Raman spectra showing the characteristic peak at 1014 cm^−1^. (**C**) SEM image of the precipitates (without inoculum); the yellow arrow points to small globules. (**D**) SEM image of *A. ferrooxidans* grown in 9K medium containing lausenite. An example of a cell (white dashed line) being covered completely by lausenite (yellow arrow). (**E**) EDS analysis of lausenite in 9K medium (without inoculum). (**F**) Moist, lumpy FeSO_4_·7H_2_O (up) versus dry FeSO_4_·7H_2_O (down).

It was important to note that precipitates may form when preparing 9K medium, which negatively affects the growth of *A. ferrooxidans*. It was observed that freshly made clear 9K medium (Figure 1A, left) turned cloudy after having stood for more than 6 h (Figure 1A, right). The precipitation was so severe that it greatly reduced light transmission, and the number written on the back of the glass container was no longer visible. Additionally, the precipitates were chemically stable and did not dissolve in 5M H_2_SO_4_. Raman spectroscopy identified the characteristic peak of the precipitates as lausenite, with the structural formula of Fe_2_(SO_4_)_3_·6H_2_O (Figure 1B). SEM revealed that the precipitates appeared as numerous small spherical particles (Figure 1C) that completely covered the rod-shaped *A. ferrooxidans* cells (Figure 1D). This wrapping affected *A. ferrooxidans*’s growth and energy exchange, the Fe^2+^ were not oxidized for cells, the medium color remained the same (Figure 1A, right) for more than 7 days, and the number of cells did not increase in the last 7 days. EDS analysis revealed that Fe, S, and O were the major elements in the precipitates (Figure 1E).

When preparing 9K medium, we recommend first adjusting the pH of 700 mL of water to 1.8 using 5M H_2_SO_4_, and then adding all of the remaining reagents of solution A at once and stirring until they are completely dissolved. Next, prepare Solution B separately by dissolving dry FeSO_4_·7H_2_O in 300 mL of water. Finally, combine Solution A and solution B and adjust the pH to 1.8 with 5 M H_2_SO_4_. This procedure effectively prevents the formation of precipitates and is better suited for culturing *A. ferrooxidans*. It is worth noting that using moist, lumpy FeSO_4_·7H_2_O (Figure 1F, up) is more likely to result in lausenite precipitation when compared to using dry FeSO_4_·7H_2_O (Figure 1F, down). As storing FeSO_4_·7H_2_O in a dry and dark environment is very critical, one should consider keeping the reagent in a desiccation chamber (as simple as a sealed box containing desiccants) in humid seasons and at humid locations such as Hainan Island.

### 3.2. Chemoautotrophic Growth of A. ferrooxidans

**Figure 2:**
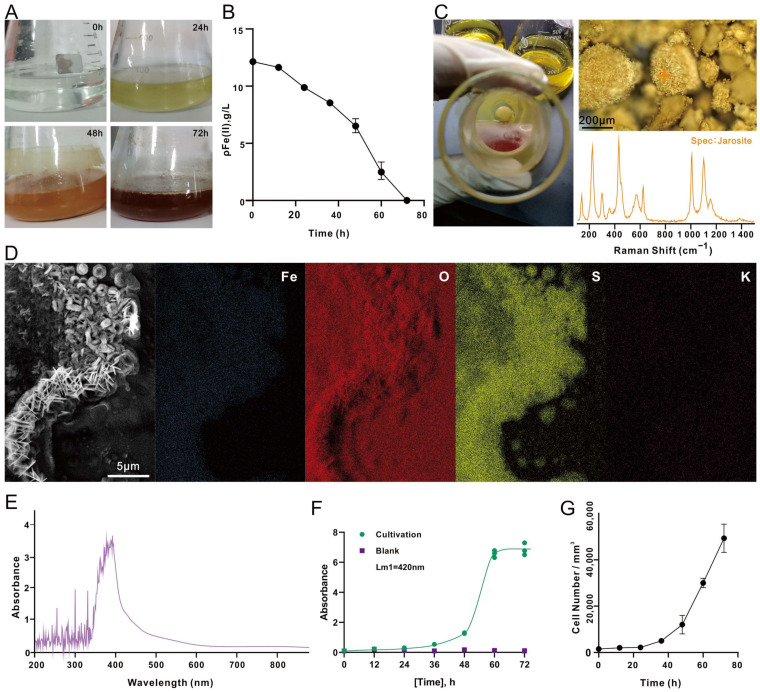
Chemoautotrophic growth of *A. ferrooxidans*. (**A**) The color of culture medium observed at different time points (0 h, 24 h, 48 h, and 72 h, respectively). (**B**) The change in Fe^2+^ concentration (ρ, g/L) in the cell culture over time. (**C**) Yellow precipitates deposited at the bottom of the flask after 48 h of incubation and the representative Raman spectra. (**D**) EDS analysis of the yellow deposits. (**E**) Full-wavelength (200–900 nm) absorbance scan of the cell culture sampled at T = 48 h. (**F**) Absorbance at 420 nm of the cell culture (green) and the blank control (purple) over time. (**G**) Cell concentration (number/mm^3^) over time determined by cell counting.

As the *A. ferrooxidans* grew, the culture medium changed color. In the beginning of cultivation (0 h), the 9K medium was light green, due to the presence of high FeSO_4_·7H_2_O concentration. As the incubation time increased, the medium gradually changed to light yellow (24 h), and then to yellowish brown (48 h), and finally, brownish red (72 h) (Figure 2A) when the Fe^2+^ level in the medium was too low to be detected (Figure 2B). After 48 h of incubation, significant amounts of yellow precipitates appeared at the bottom of the conical flask. Raman spectroscopy identified the yellow deposits as jarosite, as a complete spectrum of jarosite was observed (Figure 2C). The characteristic peaks of jarosite encompass the vibration modes of *v3*(SO_4_^2−^) at 1000–1015 cm^−1^, *v1*(SO_4_^2−^) at 1090–1115 cm^−1^ and three Fe-O at wave numbers below 440 cm^−1^ [40]. The identification of the yellow deposits was supported by SEM-EDS analysis, which detected that the elements in jarosite were mainly Fe, O, S and K (Figure 2D). The full-wavelength absorbance scan showed that the cell culture of *A. ferrooxidans* had maximum absorbance at a wavelength of 420 nm (Figure 2E); the growth of *A. ferrooxidans* was therefore recorded at OD420. The absorbance increased rapidly at 48–60 h (Figure 2F), and the increase was attributed to the rapid production and accumulation of precipitated metabolites. Cell concentration determined by cell counting indicated that the culture entered the exponential phase at 48 h and reached the maximum at the completion of the experiment (72 h), which matches the growth trend observed via OD420 (Figure 2G).

According to the chemical reactions listed for the oxidation process of Fe^2+^ by *A. ferrooxidans* [41], the conversion of Fe^2+^ to Fe^3+^ mediated by *A. ferrooxidans* occurred in the early stage (Equation (1), and the consumption of H^+^ caused the pH to increase; as the incubation continued, the Fe^3+^ ions in the solution increased, and a series of hydrolysis reactions of Fe^3+^ ions took place, which further increased the acidity of the solution (Equations (2)–(5)). Then, the chemically unstable Fe(OH)_3_ reacted with SO_4_^2−^, Fe^3+^, and NH_4_^+^ in the medium to produce yellow jarosite (Equation (6)), which was observed in significant amounts after 48 h of inoculation (Figure 2C):

4Fe^2+^ + 4H^+^ + O_2_ — *^A. ferrooxidans^* → 4Fe^3+^ + 2H_2_O (1)

4Fe^3+^ + 4H_2_O → 4Fe(OH)^2+^ + 4H^+^ (2)

4Fe(OH)^2+^ + 4H_2_O → 4Fe(OH)_2_^+^ + 4H^+^ (3)

4Fe^3+^ + 4H_2_O → 2Fe_2_(OH)_2_^4+^ + 4H^+^ (4)

2Fe_2_(OH)_2_^4+^ + 8H_2_O → 4Fe(OH)_3_ + 8H^+^ (5)

3Fe(OH)_3_ + 4SO_4_^2−^ + 3Fe^3+^ + 3H_2_O + 2NH_4_^+^ → 2M[Fe_3_(SO_4_)_2_(OH)_6_] + 3H^+^ (6)

where M can be K^+^, H_3_O^+^, or NH_4_^+^.

**Figure 3:**
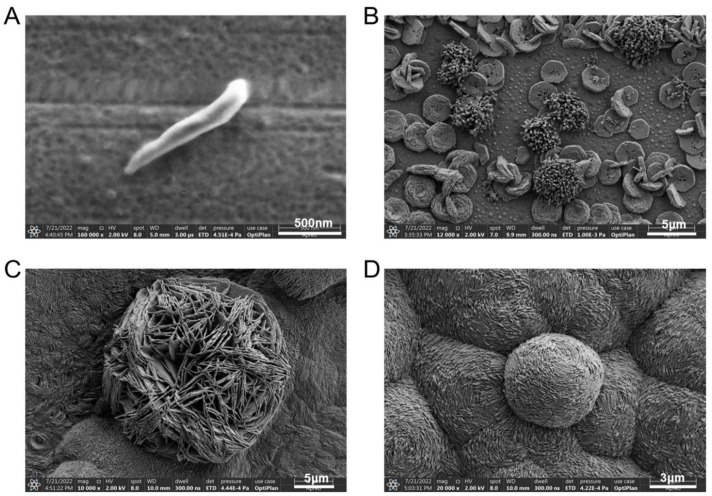
SEM images of chemoautotrophic *A. ferrooxidans* and biominerals. (**A**) An example of chemoautotrophic cell grown in correctly prepared 9K medium (without lausenite) collected at T = 48 h. (**B**) Flat round discs of jarosite and strings of iron oxides. (**C**) Iron oxides on a jarosite disc. (**D**) Starved cells without replenishing any Fe^2+^ (T = 1 month).

The *A. ferrooxidans* cells grown in the correctly prepared 9K medium (i.e., without lausenite formation) for 48 h were in rod shape (Figure 3A). Their surface was smooth and was not covered by lausenite globules (Figure 1D). We believe that these cells were in a good growth state because they would be able to fully acquire electrons and exchange small molecules with the medium. SEM analysis revealed that the jarosite and iron oxides (hematite) which appeared after 48 h of cultivation looked like flat round discs and hairy strings, respectively, which agreed with the features described in previous studies [42,43].

For some of the chemoautotrophic cultures, after having confirmed that the Fe^2+^ had been exhausted after three days of incubation (Figure 2B), we extended the cultivation period to 1 month without replenishing Fe^2+^ ions. When deprived of an energy source, *A. ferrooxidans* does not divide and undergoes morphological change. *A. ferrooxidans* cells changed from rod shape to coccoid shape, with the surface being covered by iron oxides (Figure 3D). This may be an adaptation strategy to resist adverse situations [44], such as starvation [45,46]. This suggests that microorganisms are more likely to adopt the survival–starvation law in environments lacking sufficient energy in order to survive [47].

### 3.3. Electroautotrophic Growth of A. ferrooxidans

**Figure 4:**
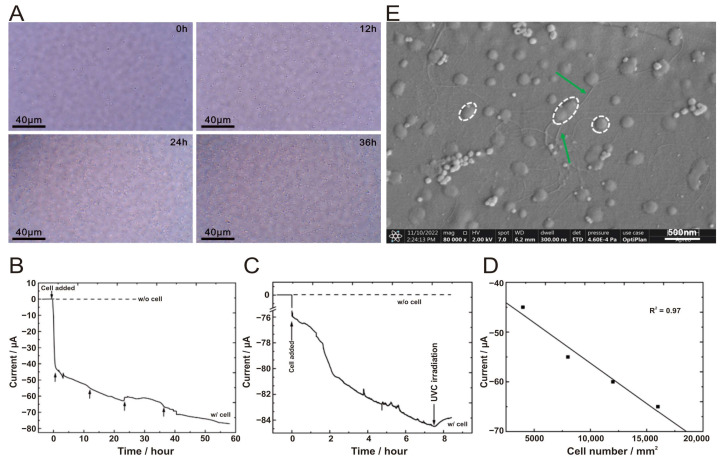
Electroautotrophic growth of *A. ferrooxidans*. (**A**) In situ Raman optical microscope (100×) observation of an FTO electrode surface was conducted at the indicated time (0 h, 12 h, 24 h and 36 h) after cell inoculation. The FTO electrode’s geometric area was 0.8 cm^2^. The initial OD_420_ was 0.02. (**B**) Current versus time measurements were taken to evaluate microbial current generation by *A. ferrooxidans* cells on a fluorine-doped tin oxide (FTO) electrode in the absence of Fe^2+^ ions (solid line) at +0.4 V (vs. SHE). Additionally, electric current versus time measurements without cells at +0.4 V are also depicted as a reference (dashed line). Arrows correspond to the time points in Figure 4A. (**C**) The effects of deep-UV (254 nm) irradiation on microbial current generation by cells in the absence of Fe^2+^ ions at +0.4 V (solid line). Current versus time measurements without cells at +0.4 V are also depicted as a reference (dashed line). (**D**) Plot of microbial current against the number of cells attached to an electrode surface obtained from in situ Raman optical microscope observation. The correlation coefficients’ squares were calculated by adding the point of origin to the obtained data. The cathodic current was plotted against the number of cells connected to the FTO working electrode to quantify their relationship. (**E**) SEM image of electroautotrophic *A. ferrooxidans* (48 h); white dashed line indicates the coccoid-shaped cells and green arrows show the conductive pilus-like structures.

The design and the small size of the BES used in this study allow for the optical imaging of the FTO-coated glass electrode surface, located horizontally at the bottom of the BES, to be obtained throughout the course of the experiment without interrupting the BES chamber. In situ microscopic observation under a bright field using the Raman spectrometer clearly showed that the number of cells on the FTO-coated glass electrode increases over time (Figure 4A).

There have been studies linking the growth of *A. ferrooxidans* to an applied electric field. It has been demonstrated that the presence of Fe^2+^ has a positive/negative effect on the ferrous oxidative metabolism of *A. ferrooxidans* when stimulated with an applied constant potential [28]. Controlling the electrochemical reduction reaction of Fe^3+^ to Fe^2+^ in the culture system by applying an electrode potential resulted in a more efficient bacterial culture. This approach led to fewer side reactions, such as hydrogen production, and an increased bacterial growth rate in the 0.72–0.22 V (vs. SHE) potential range compared to the electric current culture method [27]. When the applied constant potential was increased to 0.92 V (vs. SHE), the growth of *A. ferrooxidans* was inhibited, and prolonged high-voltage stimulation even led to the death of the bacteria below the initial inoculum concentration. This may be attributed to the fact that high field strength increases the hydrophobicity of the bacterial surface and the surface charge, which in turn inhibits the growth and reproduction of the bacterium [48]. In contrast, applying an electric field of 0.42 V (vs. SHE), stimulated changes in the oxidative electron transfer capacity of the cell membrane of *A. ferrooxidans* bacteria, ultimately accelerating their growth and metabolism [49]. Although the studies were conducted in the presence of Fe^2+^, the potential effect of current on *A. ferrooxidans* growth is still evident.

As we were inspired by the above studies, after successfully establishing a stable culture process for chemoautotrophic growth of *A. ferrooxidans*, the Fe^2+^-free electroautotrophic growth of *A. ferrooxidans* were carried out according to their work [14]. In the presence of *A. ferrooxidans* cells, the cathodic current was gradually increased to −75 μA after 60 h of incubation (the solid line in Figure 4B). The cathodic current was increased by a total of 20 µA 36 h later compared to the initial measurement (0 h), and the current draw (i.e., electron consumption) was due to the presence of cells. Conversely, constant currents were observed for the blank control (without inoculum) (the dashed line in Figure 4B), indicating that no cathodic current was generated in the absence of *A. ferrooxidans* cells. After the BES chamber was irradiated in situ with an ultraviolet lamp (254 nm) that inhibited bacterial activity, the cathodic current showed a decreasing trend (i.e., the current became less negative) (Figure 4C). This result further supports the conclusion that the metabolic activity of *A. ferrooxidans* cells is coupled with the generation of the cathodic current, possibly due to electron consumption. Furthermore, there is a strong correlation (R^2^ = 0.97) between the magnitude of the cathodic current and the number of cells attached to the FTO-coated glass electrode (Figure 4D). These data strongly suggested that the cathodic current generation was indeed a result of extracellular electron transfer to the cells directly attached to the electrode surface. Using in situ cell counting and by disabling the direct uptake of electrons from the electrode with CO treatment of viable cells under in vivo conditions, a previous study also considered the possibility that the cathodic current generated by *A. ferrooxidans* in a BES chamber with similar configuration was primarily due to the cells attached to the FTO-coated glass electrode rather than the suspended cells [14]. Indeed, we had also taken some samples from the liquid phase in the BES chamber for cell counting, yet the number of suspended cells was found to be very low or too low to be detected. As in the study by Ishii and coworkers [14], which we referenced for the BES design and the experimental setup, we did observe the same electron consumption phenomenon and cell growth trend as they did in their study. However, we noticed some differences in the magnitude and range of current and in number of cells. Such discrepancies can be attributed to the differences in the size of the FTO-coated glass electrode (0.8 cm^2^ in this study, versus 3.14 cm^2^), the initial amounts of cells in the inoculation (OD420 = 0.02 in this study, versus OD500 = 0.02), and the choice of different media that yielded different conductivity and cellular activity (3.0 g/L (NH_4_)_2_SO_4_ in the 9K medium in this study, versus 0.132 g/L (NH_4_)_2_SO_4_ in the DSMZ medium 882).

SEM images of electroautotrophic *A. ferrooxidans* after 48 h of incubation provided direct evidence that the cells were coccoid- or short rod-shaped and sprouted abundant pilus-like structures (Figure 4E), which looked different from chemoautotrophic *A. ferrooxidans* cells (Figure 3A). Moreover, the electroautotrophic cells were embedded in EPS and formed a thin biofilm (Figure 4E). Notably, these morphological changes are different from those introduced by stress due to starvation (Figure 3D), which may be indicative of the adaptive response of *A. ferrooxidans* to use extracellular electrons.

Additionally, we also calculated the relative growth rate (k) and then the doubling time (or generation time) of electroautotrophic and chemoautotrophic *A. ferrooxidans* cells using this equation: μ = (ln2)/k. The electroautotrophic cells doubled in number every 11.35 h, which is about 2.5 times longer than the time it took for chemoautotrophic cells to double once (μ = 4.46 h). Correspondingly, the observed growth rate (k = 0.06 cells/h) of the electroautotrophic cells was 2.5 times slower than that of the chemoautotrophic cells (k = 0.16 cells/h). The possible explanations for the difference in the doubling time are as follows: (1) the volume effect of the containers used for the electroautotrophic and chemoautotrophic growth experiments (5 mL versus 150 mL); (2) the bioavailability of electron donors (electrode versus dissolved Fe^2+^), O_2_ as the electron acceptor (no shaking verses shaking), and CO_2_ as the carbon source (no shaking versus shaking). Previous studies have proposed that Fe^2+^, as an electron donor for *A. ferrooxidans*, effectively activates proteins in the extracellular electron transport chain [22]. In contrast, similar information is lacking regarding the effective amounts of electrons extracted from the electrode for the synthesis of ATP molecules, and the conversion efficiency of energy from ATP consumption to biomass production. Accurate quantification of such information will be invaluable and should be pursued in future research.

### 3.4. Transcriptomic Expression of A. ferrooxidans under Electroautotrophy vs. Chemoautotrophy Conditions

**Figure 5:**
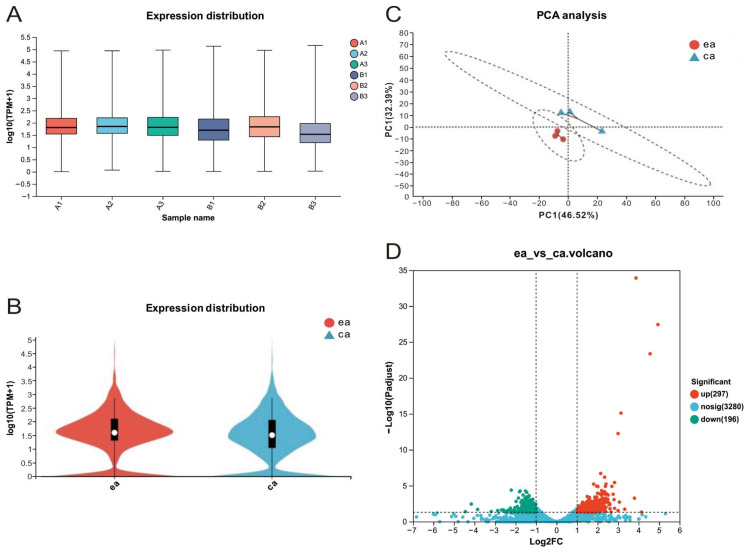
Genes expression distribution in electroautotrophic and chemoautotrophic *A. ferrooxidans*. (**A**) samples’ genes expression distribution of ea (A1, A2, A3) and ca (B1, B2, B3). The horizontal axis represents the sample names, and the vertical axis represents log_10_(TPM + 1). Each color in the figure represents one sample, and the horizontal line indicates the median gene expression in the sample. (**B**) The group expression distribution of ea (red) and ca (blue). The plot displays sample names on the horizontal axis and log_10_ (TPM + 1) on the vertical axis. Each color represents a different sample, and the inflated portion of the plot highlights the region with the highest concentration of gene expression in the entire sample. The principal component analysis of ea (red) and ca (blue) is shown in (**C**). The samples underwent dimensionality reduction using relative coordinate points on the principal components. The distance of each sample point represents the distance between the samples, with closer distances indicating higher similarity. The 2D plot distinguishes the samples based on the contribution of principal component 1 (PC1) on the horizontal axis and principal component 2 (PC2) on the vertical axis. (**D**) The volcano plot shows the differentially expressed genes (DEGs) between ea and ca, with the horizontal coordinate representing the fold change in gene expression difference between the two sets of samples (FC value). The graph displays the statistical test for the difference in gene expression change, represented by the vertical coordinate, which is the *p*-value. Higher *p*-values indicate more significant expression differences. The horizontal and vertical coordinates are in log scale. Each point on the graph represents a specific gene, with red points indicating significantly up-regulated genes, green points indicating significantly down-regulated genes, and blue points indicating non-significantly different genes. After mapping all the genes, it is evident that the genes on the left are down-regulated, while those on the right are up-regulated. The significance of the difference in expression increases as the points move towards the sides and the top.

The number of raw transcriptomic reads per sample ranged from 23,792,836 to 27,301,650. After removing low-quality reads, a total of 45.47 Gb of high-quality reads was obtained, averaging 7.58 Gb per sample. A total of 3526 genes were annotated, with 1359 genes being shared by all six samples (3 ea and 3 ca). The gene expression distribution of the six samples (A1, A2, A3, B1, B2, B3) greatly overlapped (Figure 5A), as well did that between the two groups (ea vs. ca) (Figure 5B), which are preferable for downstream statistical analysis. The PCA of the transcriptomic data clustered the 6 samples by group (Figure 5C). A total of 493 DEGs were identified using the threshold values of absolute log_2_ FC values ≥1 and adjusted *p*-values <0.05, respectively. In comparison to the chemoautotrophically (ca) expressed genes of *A. ferrooxidans* cells, 297 genes were down-regulated and 196 genes were up-regulated in electroautotrophic (ea) *A. ferrooxidans* cells (Figure 5D).

**Figure 6:**
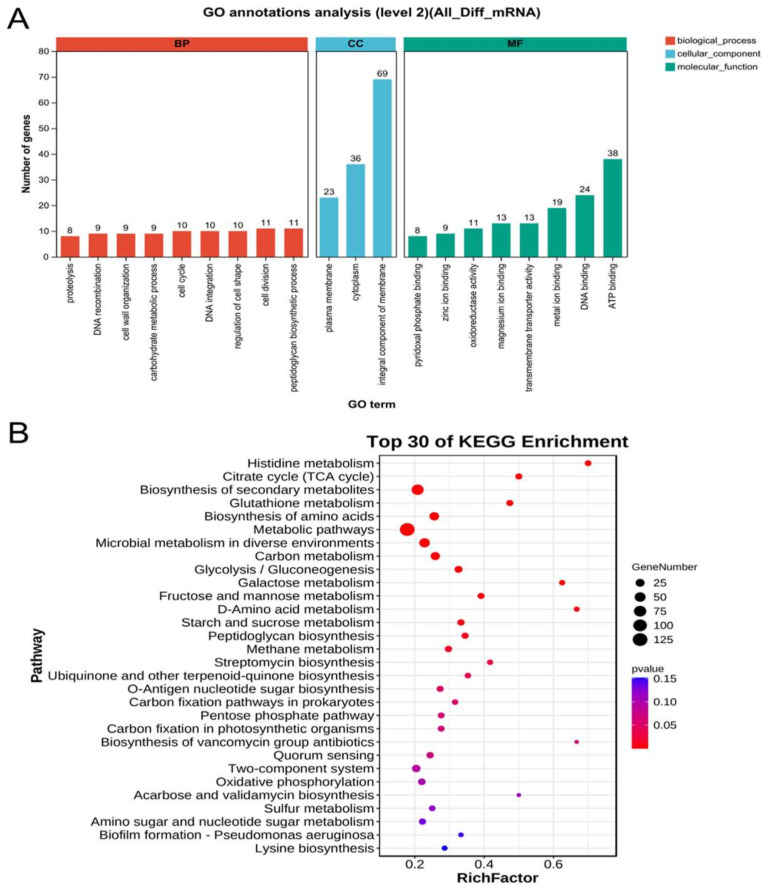
GO/KEGG enrichment and annotation analysis of DEGs. (**A**) The horizontal axis represents the secondary classification term of GO, the left vertical axis represents the percentage of the total number of genes included in that secondary classification, and the right vertical axis represents the number of genes compared to that secondary classification. The three colors indicate the three major classifications, biological process (red), cellular component (blue), and molecular function (green). (**B**) Horizontal coordinates indicate the name of the KEGG metabolic pathway, and vertical coordinates indicate the number of genes annotated to that pathway. The size of the circle represents the gene number (25–125) and the redder the color, the lower the *p* value (<0.05).

The GO and KEGG databases were used to identify the functional information of the detected DEGs. GO annotation is divided into three categories: biological process, cellular component, and molecular function. The functional description of cellular component accounted for 20% of GO annotations, with 168 genes involved in the integrative component of membrane, cytoplasm, and plasma membrane (Figure 6A). The molecular function category was dominated by genes related to ligands or chaperons that bind to ATP, DNA, and metal ions (Figure 6A). In the biological process category, genes were distributed in peptidoglycan biosynthetic process, cell division, and regulation of cell shape (Figure 6A). The DEG metabolic pathways in KEGG can be categorized into these groups: metabolism, environmental information processing, and cellular processes. DEGs are enriched in multiple pathways related to energy metabolism and cellular processes. According to the top 30 of KEGG enrichment, the genes involved in electroautotrophic growth are significant (gene number >50, *p*-value < 0.05) enriched in metabolic pathways and biosynthesis of secondary metabolites (Figure 6B).

During the chemoautotrophic growth of *A. ferrooxidans*, bc1 complex was down-regulated as expected. It has been reported that the pet I and pet II operons code for the bc1 complex, which is responsible for electron transfer in the uphill pathway of Fe^2+^ oxidation [50]. The pet I operon is expressed only when Fe^2+^ is used as an energy source and shows a decreasing trend with increasing levels of Fe^2+^ oxidation. Pet II is transiently expressed during the logarithmic phase of bacterial growth when Fe^2+^ is the only energetic substrate; however, it is continuously expressed throughout the entire period of growth when sulfur serves as an energetic substrate [51,52]. Previous research has also demonstrated that the use of different energy substrates, specifically S and Fe^2+^, can lead to distinct protein expression profiles in *A. ferrooxidans* [53]. It has been reported that the expressions of seven proteins, including rusticyanin, cytochrome c552, PstS, CbbQ protein, and the small and large subunits of ribulose biphosphate carboxylase are up-regulated when Fe^2+^ is used as the energy substrate, and these proteins have been isolated and identified [53]. Another study has shown that the expression of the *rus* operon is influenced by substrates. It is expressed continuously when Fe^2+^ donates an electron until the supply of Fe^2+^ is exhausted [54,55]. These results suggest that chemoautotrophy involves genes that are recruited differently depending on the diverse input energy sources. This result helps in understanding the transcriptomic data of electroautotrophy.

We looked up genes known to be involved in Fe^2+^ oxidation [15,16,56] in the set of DEGs. Interestingly, the genes that are essential for chemoautotrophic electron transfer are down-regulated in electroautotrophic (Fe^2+^-free) cells (Table 1). For electroautotrophy, as the source of electron has changed to solely from the electrode, the initial steps for electron acquisition would likely be different from those for chemoautotrophy. However, the downhill and uphill pathways that lead to ATP production and reductant regeneration for CO_2_ fixation, respectively, are deemed to be as critical to growth by electroautotrophy as that by chemoautotrophy. The reason why these pathways are down-regulated during electroautotrophic growth is not clear.

We then examined the up-regulated DEGs and found that many genes encoding membrane channel proteins were significantly up-regulated under electroautotrophy, including, for example, pore proteins and type IV pilin proteins (Table 2). This is very intriguing because many studies have investigated the role of type IV pili of *A. ferrooxidans* in various bacterial processes, including mineral surface attachment and colonization, twitching type motility, and electron transfer [57,58,59]. Type IV pili, also known as Tfp-pili [60], are encoded in the *pil* gene cluster of the *A. ferrooxidans* strain used in this study [10]. Conducting-probe atomic force microscope (AFM) analysis of the Tfp of *A. ferrooxidans* revealed that they are highly conductive [59]. Porins are protein molecules found in the outer membrane of Gram-negative bacteria. They form multimeric channels for the passive diffusion of water, ions, or other small molecules. Porins are beta barrel proteins that act as a pore, allowing molecules to diffuse [61]. Porins are unique among membrane transport proteins in that they act as channels that are specific to different types of molecules, allowing for passive diffusion. This means that they are large enough to allow for the movement of molecules without the need for active transport. It is important to note that porins are specific to different types of molecules. As the pilus grows, it emerges through the outer membrane via an outer membrane pore [62]. Together with pilin proteins, porins form an electron channel [63]. By utilizing pore proteins, *A. ferrooxidans* establishes a direct connection between its cellular machinery and an external iron source, facilitating the transfer of extracellular electrons from the Fe^2+^ substrate to subsequent components of the iron respiratory chain [56]. Up-regulation of type IV pili and porin proteins detected in our study indicated that they are critical for the phenomenon of electron consumption by electroautotrophic cells of *A. ferrooxidans*, as described in Section 3.3.

Electron transfer is mediated not only by pili but also by membrane protein. Transcriptomic data showed that ferredoxin is up-regulated (Table 2). Ferredoxins are a group of iron-sulfur proteins that facilitate electron transfer in various metabolic reactions. They can be classified into different subgroups based on the type of iron-sulfur cluster(s) present. One such subgroup is the 4Fe-4S ferredoxins, commonly known as ‘bacterial-type’ ferredoxins, found in bacteria [64]. The structure of these proteins comprises the duplication of a domain consisting of twenty-six amino acid residues. Each of these domains contains four cysteine residues that bind to a 4Fe-4S center [65]. This is consistent with the gene description given in Table 2.

Besides the membrane protein ferredoxins, outer membrane, inner membrane, and transmembrane proteins are also considered mediators of electron transport. The TolC outer membrane protein is a member of the envelope protein family found in Gram-negative bacteria. It is a channel protein that mediates the extracellular transport of various types of compounds [66]. ABC transporters are transmembrane proteins that use the energy from ATP hydrolysis to carry out various biological processes, including the translocation of different substrates across membranes and non-transport-related processes [67]. The major facilitator superfamily (MFS) is a group of membrane transport proteins that move small solutes across cell membranes in response to chemiosmotic gradients [68]. Molecules of this type are present in bacteria, archaea, and eukarya and include some that can function by solute uniport, solute/cation symport, solute/cation antiport, and/or solute/solute antiport with inward- and/or outward-directed polarity [69]. TonB-dependent receptors (TBDRs) facilitate substrate-specific transport across the outer membrane in Gram-negative bacteria. This transport is powered by the proton motive force transmitted from the TonB-ExbB-ExbD complex located in the inner membrane (TonB system) [70]. Additionally, TBDRs mediate the transport of siderophores into the periplasm. The process of siderophore uptake across the outer membrane is facilitated by a complex of three membrane-spanning proteins: TonB, ExbB, and ExbD. This process is coupled with the chemiosmotic potential of the cytoplasmic membrane [71]. Moreover, the MotA/TolQ/ExbB proton channel family protein appears to be involved in the translocation of proteins across a membrane and is likely a proton channel. MotA is a crucial element of the flagellar motor that employs a proton gradient to produce rotational motion in the flagellum [72]. The outer membrane, inner membrane, and transmembrane proteins mentioned above are all activated to varying degrees during extracellular electron uptake in *A. ferrooxidans* and play key roles in electrons transfer pathways.

Based on the available data, it is difficult to determine the electron acceptor(s) after the electrons have been delivered to the inner cell membrane via the above-mentioned pili and transmembrane proteins. The uphill and downhill genes are all expressed under the electroautotrophic condition, even though the genes are down-regulated. It seems that the missing link is how the import of electrons through pili and transmembrane proteins is connected to the conventional (uphill and downhill) pathways for electron transport. It is still unclear whether branching point(s) exist in the electron transport pathway of electroautotrophy that funnel electrons to the uphill and downhill pathways, similar to RUS in the chemoautotrophic mode.

Please note that the membrane proteins mentioned above, in addition to their possible involvement in electron transfer, may also be involved in cellular detoxification and stress response [73]. Previous research has demonstrated that when an electric field is applied to cells, they generate a higher transmembrane potential, which increases the glycosylation of cell membrane proteins and alters the permeability of the cell membrane [74]. We also found that the following genes were up-regulated in ea samples when compared with the samples: glycosyltransferase (AFE_RS01070, Log_2_FC = 2.80), methyltransferase (AFE_RS11125, Log_2_FC = 1.49), toxicologic efflux protein (AFE_RS15840, Log_2_FC = 2.07), osmotic regulation protein (AFE_RS14785, Log_2_FC = 1.94), and stress protein (AFE_RS10070, Log_2_FC = 1.57). Therefore, the up-regulated expression of membrane protein genes may also indicate that cellular response triggered by electroautotrophy is similar to stress response.

The transcriptomic results offer a more comprehensive explanation. The genes encoding for several proteins, including succinate--CoA ligase (sucD −1.46 down-regulated), cell division protein (ftsA −1.62 down-regulated), and DNA polymerase (polX −1.86 down-regulated) are also present in *A. ferrooxidans*. The cell wall of *A. ferrooxidans* is composed of a 2 nm-thick peptidoglycan layer [75]. The cell wall contains carboxyl groups (-COOH), amino groups (-NH_2_), and hydroxyl groups (-OH) derived from cell wall components such as lipopolysaccharides, lipoproteins, and bacterial surface proteins [76,77]. These genes involved in cell division and cell differentiation are down-regulated, suggesting that the electroautotrophic growth of *A. ferrooxidans* is slower than the chemoautotrophic growth rate, which agrees with the results of the calculated doubling time that the electroautotrophy of *A. ferrooxidans* have a longer generation time compared to that of chemoautotrophy (Section 3.3).

In summary, in the presence of an electric field, cells undergo morphological changes and exhibit altered growth behavior as previously reported [48,49,74]. It is hypothesized that the use of electrons as a direct energy supply when Fe^2+^ is not present may impact the cellular response of the cells, increasing their ability to adapt to environmental stresses and excrete toxins. However, we should keep in mind that differences in culture methods may also reduce, to some degree, the cells’ ability to divide and form cell membranes, synthesize DNA, and utilize energy.

### 3.5. Joint Analysis of A. ferrooxidans Transcriptome and Metabolome under Mlectroautotrophy vs. Chemoautotrophy

**Figure 7:**
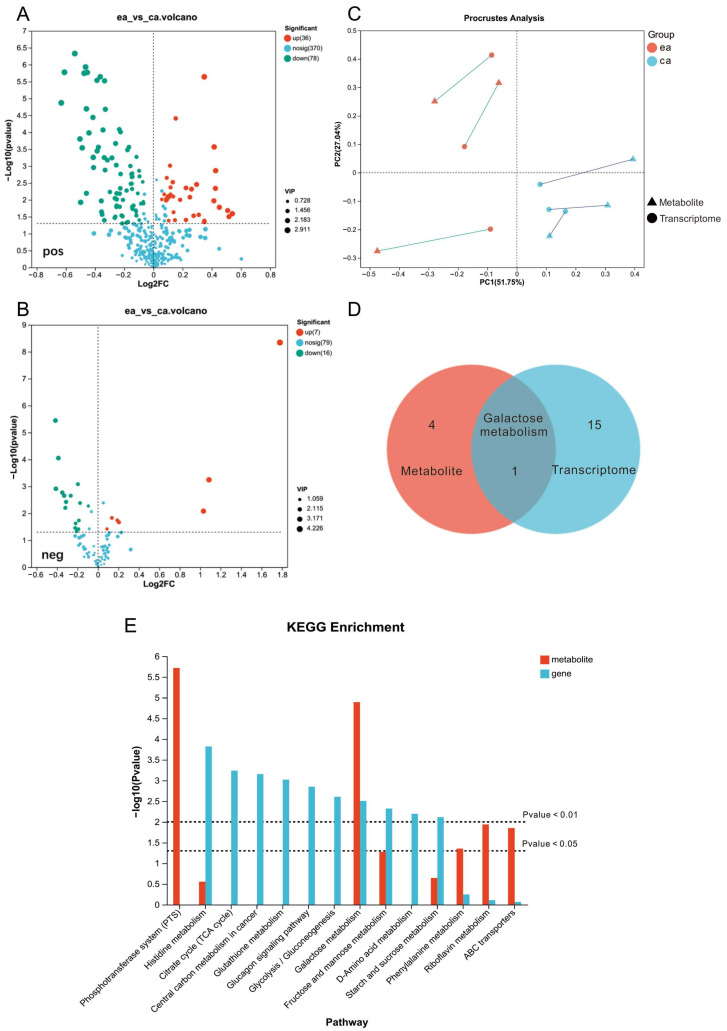
Transcriptome and metabolome conjoint analysis of ea vs. ca. (**A**) DEMs in positive ion patterns. (**B**) DEMs in negative ion patterns. (**C**) Procrustes analysis of DEGs and DEMs, sequencing analysis by PCA, to analyze the expression variability between associated features and metabolites across groups. Triangles represent metabolites and circles represent transcriptome. Red represents ca and blue represents ea. (**D**) KEGG pathway enrichment Venn diagram. Venn diagrams for pathways significantly enriched in gene set (*p* < 0.05) and pathways significantly enriched in metabolite set (*p* < 0.05). (**E**) KEGG pathway enrichment analysis of DEGs and DEMs. The pathways significantly enriched for genes in the gene set and metabolites in the metabolite set were identified using the hypergeometric distribution algorithm. The *p*-value was corrected using the BH method, and this pathway was considered significantly enriched when the corrected *p*-value (P_adjust_) was <0.05. Red color represents the results of metabolite annotation and blue color represents the results of gene enrichment. The horizontal coordinate is the KEGG pathway, and the vertical coordinate is the negative logarithm of the enrichment significance *p*-value with a base of 10. Pathways with a *p*-value of <0.05 is considered to be significantly enriched.

To explore the differences between ea and ca metabolites in *A. ferrooxidans*, we compared the abundance of metabolome. A total of 114 differentially expressed metabolites (DEMs) were identified from the positive ion pattern. Among them, 36 metabolites were up-regulated and 78 were down-regulated in ea compared to ca (Figure 7A). Fromthe negative ion pattern, a total of 23 DEMs were identified. Among them, 7 metabolites were up-regulated and 16 were down-regulated in ea compared to ca (Figure 7B). Procrustes analysis suggested that the metabolite and transcriptome datasets have high concordance with little variation (Figure 7C). Galactose metabolism is the only common pathway between metabolites and transcriptome that was enriched. (Figure 7D). In addition to galactose metabolism, the metabolite pathways showing high enrichment under electroautotrophy include riboflavin metabolism, phenylalanine metabolism, ABC transporters, and the phosphotransferase system (PTS). Conversely, the KEGG-enriched pathways that were highly enriched in the transcriptomic data include fructose and mannose metabolism, peptidoglycan biosynthesis, histidine metabolism, starch and sucrose metabolism, and glycolysis/gluconeogenesis categories and have a greater number of genes than other categories (Figure 7E). One of the possible reasons to explain the very few numbers of pathways being identified in both the DEGs and DEMs is that changes in metabolites may lag behind the faster and more transient changes in RNA transcripts. As a result, metabolome and transcriptome may not share common trends and enrichment pathways.

We observed that *A. ferrooxidans* cultured in BES for 48 h adhered more to the bottom of the BES compared to the chemoautotrophic culture (with/without shaking), which is probably related to the enriched galactose metabolism; the FTO electrode surface exhibited a smooth, membrane-like structure. It was reported that after 36 h of incubation with direct current (20 mA) applied, the cell membrane morphology of *Thiobacillus ferrooxidans* bacteria became thicker in the treated group than in the control group [78]. Moreover, microorganisms typically grow in a biofilm lifestyle, where adhesion between carbohydrates, proteins, lipids, eDNA, etc., and the cellular membrane is mediated by biofilms and EPS. Biofilms and EPS mediate cell adhesion to substrates and protect against harsh conditions such as desiccation or oxidative stress [79]. EPS production is a strategy employed in the process of microbial adaptation [80]. A previous study compared the effects of adding galactose and high initial Fe^2+^ concentrations as inducers for EPS production in planktonic cells of *Leptospirillum ferrooxidans* [81]. They found that *L. ferrooxidans* produced higher amounts of total EPS as the substrates (galactose or Fe^2+^) increased. The results showed that the EPS induced by galactose appeared to be more adhesive than the one induced by Fe^2+^. Other studies have shown that cells of *A. ferrooxidans* excrete significantly more EPS when cultivated on mineral substrate pyrite, compared to cells cultivated on Fe^2+^ [82,83,84,85]. Therefore, it is hypothesized that in electroautotrophic mode, where the FTO-coated glass electrode replaces the mineral substrate, *A. ferrooxidans* is more likely to colonize the electrode surface, form an EPS to immobilize the cells, and grow. The direct supply of electrons and the absence of Fe^2+^ may stimulate the generation of EPS, similar to a survival strategy to adapt environmental stresses. It has been reported for other microorganisms that EPS plays a significant role in extracellular electron uptake [86], and it can improve the efficiency of electron transport [87]. This is consistent not only with our transcriptome and metabolome joint analysis data, but also with the SEM image (Figure 4E).

Currently, our knowledge of the *A. ferrooxidans*’s metabolome is limited. No metabolomic data of *A. ferrooxidans* are available, nor co-expression data of transcriptomic and metabolomic data. A glimpse of the metabolites of *A. ferrooxidans* is restricted to biosynthesis. For instance, *A. ferrooxidans* can derive metabolic energy from the catabolic process of iron oxidation. The Fe^2+^/Fe^3+^ redox couple has the potential to efficiently drive the metabolism for microbial reduction of CO_2_ using renewable energy [25,59]. Studies have reported genetic modification of *A. ferrooxidans* to produce exogenous chemicals such as isobutyric acid (IBA) [88] or heptadecane [89,90] from CO_2_ [88]. With more metabolic data in the future, reanalysis of the current data may bring more insights into electroautotrophy and chemoautotrophy of *A. ferrooxidans*.

To sum up, in the absence of Fe^2+^ as the sole energy source, up-regulation of genes and metabolites on the galactose metabolism pathway occurs. This induces the formation of more EPS, resulting in stronger adhesion to the solid electrode surface. The increased EPS production could be interpreted as a sign that using extracellular electrons as the only energy source to support the growth of *A. ferrooxidans* may not be optimal for the cells. Switching to different energy sources (chemotrophy to electrotrophy) may have negative effects on the cells, forcing them to adjust their survival strategies by physiological and metabolic adaptations.

### 3.6. A Model of Extracellular Electron Uptake for A. ferrooxidans Electroautotrophy vs. Chemoautotrophy

Previous studies have shown that the rate of electron transfer from Cyc1 to cytochrome c oxidase is extremely slow in vitro [91,92]. Bioinformatics analysis and transcriptome data indicate that the transferring of electron that derive from Fe^2+^ oxidation to final acceptors should not be inhibited by the availability of RUS in these electron transfer chains; therefore, the existence of multiple electron transfer chains regarding Fe^2+^ oxidation is feasible [30]. It is generally believed that when Fe^2+^ is present, the extracellular electron transfer of *A. ferrooxidans* could be achieved through a dedicated redox protein on the cell outer surface. Such redox-active enzymes are often cytochromes c (Cyt c) [93,94]. Additionally, Type IV pili from *Geobacter sulfurreducens* [95] and outer membrane and periplasmic extensions from *Shewanella oneidensis* [96,97] can form highly conductive biological ‘nanowires’ for long-distance electron transfer through direct physical contact [98]. This method of long-distance electron transfer via pili may also work for *A. ferrooxidans*.

By combining transcriptomic and metabolomic data, it is suggested that in the absence of Fe^2+^ and with extracellular electrons as the sole source of energy, extracellular electron transfer in *A. ferrooxidans* may use pilin proteins to a greater extent. This process relies on pore proteins located in the cellular membrane or associated transporter proteins spanning the intracellular and extracellular membranes, as well as interstitial haemoglobin to transfer electrons. Simultaneously, the cell membrane’s permeability is altered, resulting in an increased efflux of toxicants. Additionally, galactose metabolism is enhanced, and the cells produce more EPS, enabling it to adhere firmly to the electrode and grow. EPS also plays a crucial role in the extracellular electron uptake.

**Figure 8:**
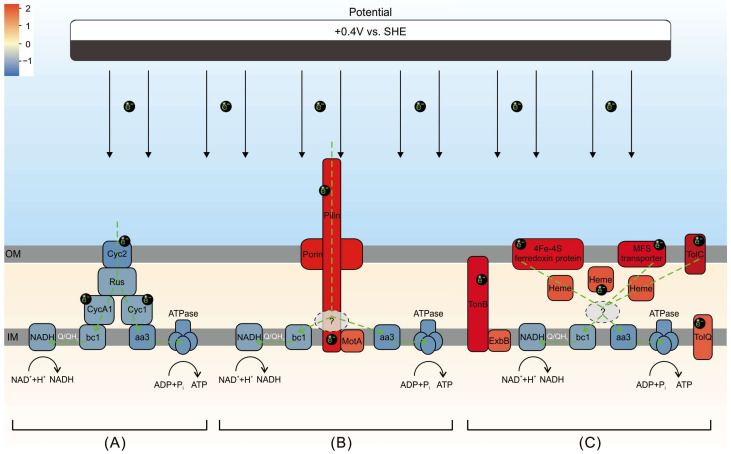
Extracellular electron uptake model of electroautotrophy (vs. chemoautotrophy). The color indicates log_2_FC in differential expressed genes, with blue indicating down-regulation and red indicating up-regulation. The grey dashed boxes represent the potential branch points. The green dashed lines indicate electron transfer pathways. (A), (B), and (C) represent extracellular electron uptake pathways that are simultaneously employed during electroautotrophic growth of *A. ferrooxidans*.

Based on the information presented, we propose an extracellular electron uptake model for electroautotrophy (vs. chemoautotrophy) (Figure 8). The figure highlights at least two key points. Firstly, as for the electroautotrophic growth, it involves most of the functional proteins that are part of the electron transport chain during *A. ferrooxidans* chemoautotrophic growth, except CycA1 and Cyc1, can be found in DEGs. Interestingly, all of these proteins appear to be down-regulated in ea vs. ca. Secondly, pilin proteins, porin proteins, ferredoxin proteins, and transporter proteins located in the outer membrane (TolC, MFS transporter), periplasm (heme), inner membrane (ExbB, MotA, TolQ), and transmembrane (TonB) are all up-regulated (ea vs. ca).

The results indicate that the expression and activity of functional proteins associated with chemoautotrophy are decreased to varying degrees when extracellular electrons serve as the sole energy source. However, the expression and activity of proteins associated with conductive pilin and transmembrane transport, which are in direct physical contact with the electrodes, are increased. This could represent additional electron acquisition pathways during electroautotrophy of *A. ferrooxidans*. To the best of our knowledge, this phenomenon has not been reported for *A. ferrooxidans* in previous studies. Regarding the downstream acceptor(s) of electroautotrophically acquired electrons from the inner membrane, the current data cannot provide a clear explanation. It is speculated that the electrons will be used partly downhill for ATP synthesis and partly uphill for the maintenance of NADH, similar to that during chemoautotrophic growth.

It is still unclear whether electrons obtained by EEU would be diverted to both conventional uphill and downhill pathways. If yes, what would be the triage protein(s), the mechanism linking to the conventional intracellular electron transfer pathways, and the electron bifurcation proportion? If no, which of the up/downhill pathways do the EEU electrons go? Is there a possibility that electrons might not be delivered to the inner cell membrane in both directions at the same time, but instead, having a bias against one of pathways? Imagine if the EEU electrons are not connected to reducing the generation of NADH and thus CO_2_ fixation or their flow is extremely limited, this could slow down the growth rate and lead to an increase in the doubling time during electroautotrophic growth compared to that during chemoautotrophic growth. Notably, we did find gene up-regulation that are related to ATP production (Table 2), such as ATP-binding cassette domain-containing protein (AFE_RS13600, Log_2_FC = 2.24), ATP-binding protein (AFE_RS07005, Log_2_FC = 1.72), and putative ABC transporter ATP-binding protein (Log_2_FC = 2.59), but no further information about their exact mechanism is available. When would these ATPases be utilized? Are these ATPase components part of A-, V- and F-types ATPases used in chemoautotrophy? Or are these specifically related to handling EEU electrons? To answer these questions, more work and effort will be required.

## 4. Conclusions

In summary, *A. ferrooxidans* can use not only the oxidation of Fe^2+^ to gain electrons to support cell growth but also the electrons provided by the solid electrode as the sole source of energy for growth and metabolism. Compared to genes essential for the chemoautotrophic mode, these genes showed a decreased expression in the condition of electroautotrophy. Conversely, genes related to cell membrane and transmembrane proteins with direct electron transfer capacity, such as pilin and porin, showed an increased expression. The morphology of the cells changed from rod-shaped to short rod-shaped or spherical, and cells developed numerous conductive pilus-like structures. Additionally, the electroautotrophic cells produced large amounts of EPS to facilitate EEU. Electroautotrophic EEU pathways are more diverse. Further research is required to understand where the EEU electrons go after being transferred to the inner membrane of the cell, and how they are transferred to the final electron acceptor, O_2_, or to the reducing power to fix CO_2_. Although direct electron energy may not be the preferred choice for *A. ferrooxidans* when Fe^2+^, reduced sulfur monomers, and other energy sources coexist, our results provide useful hints for extracellular electron use as an adaptive survival strategy in extreme environments on Earth (including early Earth) and even beyond Earth.

## Figures and Tables

**Table 1:** Differentially expressed genes involved in chemoautotrophic growth and expression values ea vs. ca (down-regulated).

Gene_id	Gene Name	Gene Description	Log_2_FC (ea/ca)	Pathways
AFE_RS16265	AFE_RS16265	NAD(P)-dependent oxidoreductase	−2.25	uphill
AFE_RS03815	AFE_RS03815	FAD binding domain-containing protein	−2.05	uphill
AFE_RS14440	*cyc2*	c-type cytochrome	−1.57	downhill/uphill
AFE_RS04465	*cydB*	cytochrome d ubiquinol oxidase subunit II	−1.54	uphill
AFE_RS14290	AFE_RS14290	FAD/NAD(P)-binding oxidoreductase	−1.54	uphill
AFE_RS00620	AFE_RS00620	cytochrome c oxidase assembly protein	−1.52	downhill
AFE_RS12010	*nuoL*	NADH-quinone oxidoreductase subunit L	−1.44	uphill
AFE_RS14425	AFE_RS14425	cbb3-type cytochrome c oxidase subunit I	−1.43	downhill
AFE_RS12615	*hslU*	ATP-dependent protease ATPase subunit HslU	−1.38	downhill
AFE_RS04470	AFE_RS04470	cytochrome ubiquinol oxidase subunit I	−1.36	uphill
AFE_RS00650	AFE_RS00650	FAD-binding protein	−1.34	uphill
AFE_RS01260	AFE_RS01260	FAD-dependent oxidoreductase	−1.26	uphill
AFE_RS12210	*apbC*	NADH:ubiquinone oxidoreductase (complex I)	−1.25	uphill
AFE_RS04275	*clpX*	ATP-dependent Clp protease ATP-binding subunit ClpX	−1.24	downhill
AFE_RS14500	AFE_RS14500	FMN-binding negative transcriptional regulator	−1.22	uphill
AFE_RS02000	*icd*	NADP-dependent isocitrate dehydrogenase	−1.22	uphill
AFE_RS14415	*rus*	Rusticyanin	−1.21	downhill/uphill
AFE_RS14430	AFE_RS14430	cytochrome c oxidase subunit II	−1.17	downhill
AFE_RS12530	AFE_RS12530	cytochrome bc complex cytochrome b subunit	−1.16	uphill
AFE_RS13940	*hisG*	ATP phosphoribosyltransferase	−1.12	downhill
AFE_RS10975	AFE_RS10975	FAD-dependent monooxygenase	−1.07	uphill
AFE_RS03470	*thyX*	FAD-dependent thymidylate synthase	−1.05	uphill
AFE_RS14650	AFE_RS14650	ATP-binding protein	−1.01	downhill

**Table 2:** Differentially expressed genes and expression values of ea vs. ca (up-regulated).

Gene_id	Gene Name	Gene Description	Log_2_FC (ea/ca)
novel0441	-	TolC family protein, partial	3.32
novel0021	-	membrane protein, putative	3.06
novel0531	-	putative ABC transporter ATP-binding protein	2.59
novel0233	-	type IV pilin biogenesis protein, putative, partial	2.50
AFE_RS00795	AFE_RS00795	MFS transporter	2.38
novel0534	-	TonB-dependent receptor	2.37
AFE_RS10580	AFE_RS10580	biopolymer transporter ExbD	2.36
novel0363	-	MULTISPECIES: 4Fe-4S dicluster domain-containing protein	2.27
AFE_RS10585	AFE_RS10585	energy transducer TonB	2.20
AFE_RS13360	AFE_RS13360	porin	2.19
novel0120	-	MULTISPECIES: MFS transporter	2.09
novel0582	-	MULTISPECIES: TonB-dependent receptor	2.01
novel0397	-	TonB family protein	1.91
novel0535	-	MULTISPECIES: energy transducer TonB	1.89
novel0185	-	heme-utilization protein HutZ, putative	1.75
AFE_RS10575	AFE_RS10575	MotA/TolQ/ExbB proton channel family protein	1.66
novel0005	-	MULTISPECIES: ferredoxin family protein	1.57
AFE_RS09710	AFE_RS09710	ABC-2 transporter permease	1.49
AFE_RS10630	AFE_RS10630	sugar porter family MFS transporter	1.28
AFE_RS13730	AFE_RS13730	peptide ABC transporter substrate-binding protein	1.19

## Data Availability

Raw transcriptome data of *Acidithiobacillus ferrooxidans* have been deposited on NCBI (https://www.ncbi.nlm.nih.gov) under BioProject ID PRJNA1078144. BioSample accession numbers: SAMN39992291-SAMN39992293 for (ea samples), and SAMN39992294-SAMN39992296 for (ca samples). Raw metabolome data of *Acidithiobacillus ferrooxidans* have been deposited on MetaboLights (www.ebi.ac.uk/metabolights) under the accession number MTBLS9595.
